# Cerebral Small Vessel Disease (CSVD) – Lessons From the Animal Models

**DOI:** 10.3389/fphys.2019.01317

**Published:** 2019-10-24

**Authors:** Muzaimi Mustapha, Che Mohd Nasril Che Mohd Nassir, Niferiti Aminuddin, Amanina Ahmad Safri, Mazira Mohamad Ghazali

**Affiliations:** ^1^Department of Neurosciences, School of Medical Sciences, Universiti Sains Malaysia, Kubang Kerian, Malaysia; ^2^Department of Basic Medical Sciences, Kulliyyah of Pharmacy, International Islamic University Malaysia, Kuantan, Malaysia

**Keywords:** cerebral small vessel disease (CSVD), animal models, biomarkers, systems biology, therapeutic

## Abstract

Cerebral small vessel disease (CSVD) refers to a spectrum of clinical and imaging findings resulting from pathological processes of various etiologies affecting cerebral arterioles, perforating arteries, capillaries, and venules. Unlike large vessels, it is a challenge to visualize small vessels *in vivo*, hence the difficulty to directly monitor the natural progression of the disease. CSVD might progress for many years during the early stage of the disease as it remains asymptomatic. Prevalent among elderly individuals, CSVD has been alarmingly reported as an important precursor of full-blown stroke and vascular dementia. Growing evidence has also shown a significant association between CSVD’s radiological manifestation with dementia and Alzheimer’s disease (AD) pathology. Although it remains contentious as to whether CSVD is a cause or sequelae of AD, it is not far-fetched to posit that effective therapeutic measures of CSVD would mitigate the overall burden of dementia. Nevertheless, the unifying theory on the pathomechanism of the disease remains elusive, hence the lack of effective therapeutic approaches. Thus, this chapter consolidates the contemporary insights from numerous experimental animal models of CSVD, to date: from the available experimental animal models of CSVD and its translational research value; the pathomechanical aspects of the disease; relevant aspects on systems biology; opportunities for early disease biomarkers; and finally, converging approaches for future therapeutic directions of CSVD.

## Introduction

Cerebral small vessel disease (CSVD) refers to a diverse range of clinical and neuroimaging findings resulting from pathological changes of various etiologies affecting the cerebral small vessels, particularly small veins, venules, capillaries, arterioles, and small arteries (Pantoni and Gorelick, 2014). More prevalent in the elderly, CSVD doubled the risk of stroke (Bernick et al., 2001; Vermeer et al., 2007) and has been shown to be responsible for about 30% of ischemic strokes (Warlow et al., 2003; Pantoni, 2010; Bath and Wardlaw, 2015). Importantly, CSVD is recognized as an important cause of cognitive dysfunction, dementia, and functional disability among the sufferers (Pantoni and Gorelick, 2014; Zwanenburg and van Osch, 2017). In fact, a recent systematic review had concluded that some neuroimaging features of CSVD are associated with an increased risk of Alzheimer’s disease (AD), a disease clinically characterized by cognitive dysfunction and dementia. However, the causal link between the two diseases remains inconclusive (Liu et al., 2018).

Notably, the inconsistency in terms of the definition, unstandardized neuroimaging reporting, and silent nature of the disease at the early stage hampers a deeper understanding of its pathogenesis and subsequent effective therapeutic measures (Wardlaw et al., 2013a). These challenges had instigated efforts among researchers to establish a standard framework in the CSVD research field. In a recent development, a standard approach in reporting neuroimaging findings had been proposed in CSVD based on the Standards for Reporting Vascular changes in neuroimaging (STRIVE) (Wardlaw et al., 2013b). In particular, the STRIVE collaborative group had advised the minimum standard requirement for image acquisition and analysis, a scientific reporting standard technique for neuroimaging features of CSVD, and suggested common terms and definitions for neuroimaging changes found in CSVD, namely (i) white matter hyperintensity of presumed vascular origin; (ii) lacunae of presumed vascular origin; (iii) recent small subcortical infarct; (iv) perivascular space; (v) cerebral microbleed; and (vi) brain atrophy (Wardlaw et al., 2013b). Nonetheless, notably, due to the current limitation of standard neuroimaging techniques, the term CSVD reflects only the neuroradiological changes of the brain parenchyma rather than the small vessels of interest (Pantoni and Gorelick, 2014). Meanwhile, postmortem examinations of the diseased small vessels revealed distinct histopathological changes, such as fibrinoid necrosis, arteriosclerosis, and atherosclerosis (Lammie, 2002; Pantoni and Gorelick, 2014).

In view of the social and healthcare burden it may incur, contemporary and collaborative efforts are necessary to generate and expand our current understanding of CSVD from the aspects of its pathomechanism, systems biology, opportunities for early disease biomarkers, and potential therapeutic approaches. This chapter summarizes these core topics from the perspectives of numerous experimental animal models of CSVD.

## Cerebral Small Vessel Disease (CSVD) – Classification and Pathogenesis

One of the predominant types of strokes resulting from the occlusion (ischemia) of small blood vessels deep within the brain is an ischemic stroke (Rouhl et al., 2009; Smith, 2017). About 30% of ischemic or lacunar strokes are thought to be due to CSVD (Rouhl et al., 2009; Patel and Markus, 2011; Heye et al., 2015).

### Definition

The definition of CSVD remains contentious due to its complex and overlapping pathophysiological mechanism. However, it is generally accepted that CSVD is mainly due to the pathological consequences of small vessel disease on the brain parenchyma rather than the underlying diseases of the vessels (Wardlaw et al., 2013c).

**FIGURE 1:**
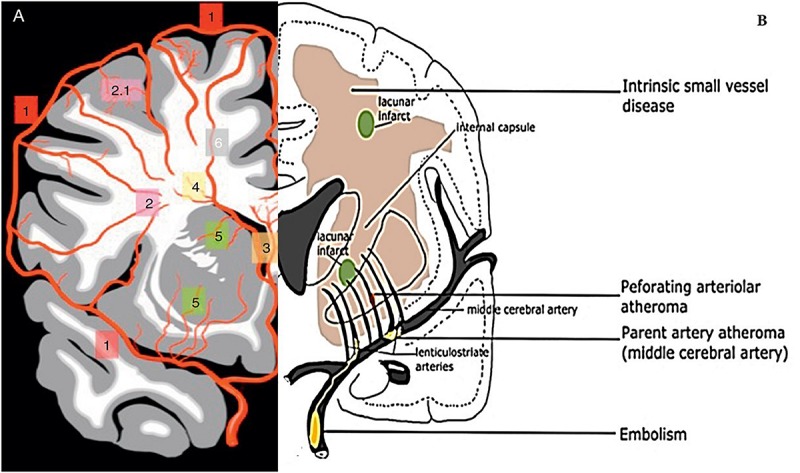
Illustration of cerebral vasculature and vasculo-pathological process of CSVD. **(A)** Different branches of cerebral arteries and their territories that supply cerebral white matter. (1) represent cortical arteries, (2) pial arterioles that supply deep white matter, (2.1) short branches, (3) anterior choroidal arteries that branch into sub-ependymal arteries, (4) arterioles of sub-ependymal, (5) MCA branches into thalamic and lenticulostriate perforating arteries that supply basal ganglia (Image source: Martorell et al., 2012). **(B)** Illustration of general aetiophatogenic features of CSVD. The picture shows branches of MCA that penetrate the subcortical region of white matter and gray matter. Embolus or thrombus may accumulate and cause occlusion (atheroma) upon the parent MCA and penetrating arteriolar. The occlusion of perforating arteriolar can cause ischemia and eventually a lacunar infarct may be formed. The core infarcts might affect surrounding tissue (penumbra). Diffused disruption of the BBB following intrinsic CSVD also occurred at the arteriolar level (Image source: Shi and Wardlaw, 2016).

Therefore, the term CSVD is preferred to describe a brain parenchyma injury that is associated with distal leptomeningeal and intracerebral vessel pathology that resides in poorly collateralized subcortical gray and deep white matter. Moreover, it is mainly due to several vasculo-pathological processes that affect and cause occlusion to the small perforating cerebral capillaries (of sizes 50–400 μm), small arteries (mostly branches of middle cerebral arteries [MCAs]), arterioles, and venules that penetrate and supply the brain subcortical region (Pantoni, 2010; Novakovic, 2010; Hinman et al., 2015; Benjamin et al., 2016) (Figure 1). Several manifestations of CSVD can be seen through clinical, radiological, or pathological phenomena with various etiologies (Ogata et al., 2014; Sorond et al., 2015; Yakushiji et al., 2018).

### Classification

There are several etiopathogenic classifications of CSVD. However, the most prevalent forms of CSVD are amyloidal CSVD (sporadic and hereditary cerebral amyloid angiopathy [CAA]) and non-amyloidal CSVD (age-related and vascular risk-factor-related small vessel, i.e., arteriolosclerosis) (Pantoni, 2010). Other less common forms of CSVD include inherited or genetic CSVD that is recognizably different from CAA (i.e., Fabry’s disease and cerebral autosomal dominant arteriopathy with subcortical ischemic strokes and leukoencephalopathy [CADASIL]), inflammatory and immunologically mediated CSVD, venous collagenosis, and other CSVD (i.e., non-amyloid microvessel degeneration in AD and post-radiation angiopathy). Table 1 describes the two major etiopathogenic classes of CSVD based on clinical and neuroimaging characteristic differences.

**TABLE 1:** Etiopathogenic classification based on clinical and neuroimaging characteristic differences in two major classes of CSVD (Vinters, 1987; McCarron and Nicoll, 2004; Love et al., 2009; Pantoni, 2010; Biffi and Greenberg, 2011; Charidimou et al., 2012; Charidimou and Jäger, 2014; Cuadrado-Godia et al., 2018; Li et al., 2018).

**Classification**	**Characteristics**	**Pathology**	**Neuroimaging features**	**Clinical syndromes**
Form 1	• Non-amyloidal CSVD • Arteriolosclerosis (age-related and vascular risk-factor-related small vessel diseases) • Advances with age • Degenerative microangiopathy	• Loss of smooth muscle cells from the tunica media (i.e., arteriolosclerosis) • Deposit of fibro-hyaline material (i.e., lipohyalinosis) • Narrowing of lumen (i.e., microatheroma) • Thickening of vessel wall (i.e., microaneurysms) • Segmental arterial disorganization	• Deep cerebral microbleeds • Rare cortical superficial siderosis • Basal ganglia perivascular space • Non-specific cerebral region of WMHs	• Lacunar strokes • Often deep (basal ganglia, thalamus, pons, cerebellum ICH) • Cognitive impairment and dementia
Form 2	• Amyloidal CSVD • Sporadic and hereditary CAA • Advances with age	• Accumulation of amyloid-β (Aβ) in the cortical walls (type 1) and leptomeningeal small arteries, but not capillaries (type 2) due to vascular occlusion and rupture • Vasculopathy (i.e., fibrinoid necrosis, loss of smooth muscle cells, wall thickening, perivascular blood breakdown, and microaneurysm) • APOE gene polymorphism (i.e., APOE ε2 and APOE ε4 allele related to types 2 and 1, respectively)	• Lobar cerebral microbleeds • Most significant feature (marker of CAA): cortical superficial siderosis • Centrum semiovale perivascular space • Posterior dominance WMHs	• Lobar ICH • Non-lacunar strokes • Transient focal neurological episodes, cognitive impairment, and dementia • Hallmarks of AD

*AD, Alzheimer’s disease; APOE, apolipoprotein E; CAA, cerebral amyloid angiopathy; CSVD, cerebral small vessel disease; ICH, intracerebral hemorrhage; WMHs, white matter hyperintensities.*

### Dynamic Pathological Processes of CSVD

In general, the various pathological changes of CSVD not only resulted in cerebral parenchyma damage, that is, axonal injury, neuronal apoptosis, demyelination, and oligodendrocyte damage (see Table 1) but also gave rise to neurological symptoms and signs, and diverse findings on neuroimaging (Li et al., 2018).

Nonetheless, the underlying pathomechanism of CSVD remains contentious despite the growing insights from histopathological, epidemiological, and physiological studies. Moreover, there is increasing evidence that advanced age and the presence of chronic hypertension may reduce the ability to self-regulate cerebral blood flow (cBF) in response to various systemic blood pressure levels and increased arterial stiffness, hence the increased speed and flow pulsatility in cerebral arterioles (Cuadrado-Godia et al., 2018). These hemodynamic changes may lead to endothelial damage in the blood–brain barrier (BBB) and alter its permeability through an increase of the shear stress (Zhang et al., 2017). Hence, the BBB breakdown is thought to be one of the major features of CSVD (Huisa et al., 2015; Wardlaw et al., 2017; Zhang et al., 2017).

Another key factor thought to contribute to the pathogenesis of CSVD is endothelial dysfunction, with elevated biomarkers being reported (Farrall and Wardlaw, 2009; Poggesi et al., 2016). In addition to the endothelium, cross-talk among cellular components of the BBB, such as pericytes, astrocytes, and oligodendrocyte precursor cells (OPCs), may be involved in the microvascular damage as precursors for the onset and progression of CSVD (Ihara and Yamamoto, 2016; Rajani and Williams, 2017). In relation to this, reduced white matter integrity due to changes in oligodendrocytes has been shown in CSVD, whereby the endothelial cell (EC)–OPC signaling became compromised and altered the ECs’ ability to secrete the releasing factor crucial for the growth and survival of OPCs to eventually cause oligodendrocytes prone to damage (Rajashekhar et al., 2006). Therefore, the interaction of multiple BBB components may play a crucial role in the discovery and development of new prevention steps and therapies for CSVD.

On the other hand, hypoperfusion or reduced cBF in CSVD has been hypothesized to be involved with endothelial dysfunction (Armulik et al., 2005). Generally, the regulation of cBF is mediated by nitric oxide (NO) signaling; thus, NO serves as a marker for endothelial dysfunction (Deplanque et al., 2013). Moreover, endothelial dysfunction was also associated with increased BBB permeability, which led to brain parenchyma lesions and worsened white matter lesions due to the reduced integrity of ECs (Young et al., 2008). Therefore, increased BBB permeability, reduced cBF, and impaired cerebral autoregulation are thought to be the major precursors to the development and progression of CSVD, although another/other potential player/s is/are still being sought.

Moreover, a group of genetically inherited forms of CSVD with an increasing prevalence has been widely investigated for the past decade (Hara et al., 2009; Pantoni, 2010). Although the molecular mechanisms underlying this form of CSVD are unclear, a multitude of studies on the monogenic form of CSVD (i.e., CADASIL) and sporadic CSVD offer new insights into the CSVD pathomechanism. Chronic cerebral hypoperfusion (CCH) and reduced cBF that lead to vascular reactivity alterations and white matter metabolic vulnerability were reported previously as markers of the inherited form of CSVD (Itoh et al., 2001; Moore et al., 2003; Hilz et al., 2004; Li et al., 2018).

Interestingly, the impaired function of the extracellular matrix (ECM) has been linked as a common disease pathomechanism between different types of monogenic CSVD, largely from proteomic and biochemical studies on postmortem monogenic CSVD in humans and animals. Moreover, increasing evidence from genetic studies supports the fact that CSVD can be highly heritable, especially among patients with early onset CSVD and young patients with stroke, and that common variants in monogenic CSVD genes may contribute to the disease pathomechanism in certain CSVD forms (Tan et al., 2017; Li et al., 2018). In addition, the increased expression of the mutated *NOTCH3* gene (a genetic determinant of CADASIL) in pericytes was found to contribute to CSVD pathogenesis due to abnormal cross-talk between ECs and pericytes (Armulik et al., 2005).

Further deliberation of the current various pathogenesis of CSVD as highlighted in the foregoing paragraphs will be discussed in the later section. Our current knowledge of the natural history of CSVD had been based largely on our neuroimaging findings, although it is limited. Recognizing the heterogeneous manifestations of CSVD, from silent to symptomatic, implies that our apparent detection of the disease is made possible through the imaging of the brain white matter. Therefore, neuroimaging remains the key modality in assessing and diagnosing CSVD.

### Neuroimaging Correlates of CSVD

**FIGURE 2:**
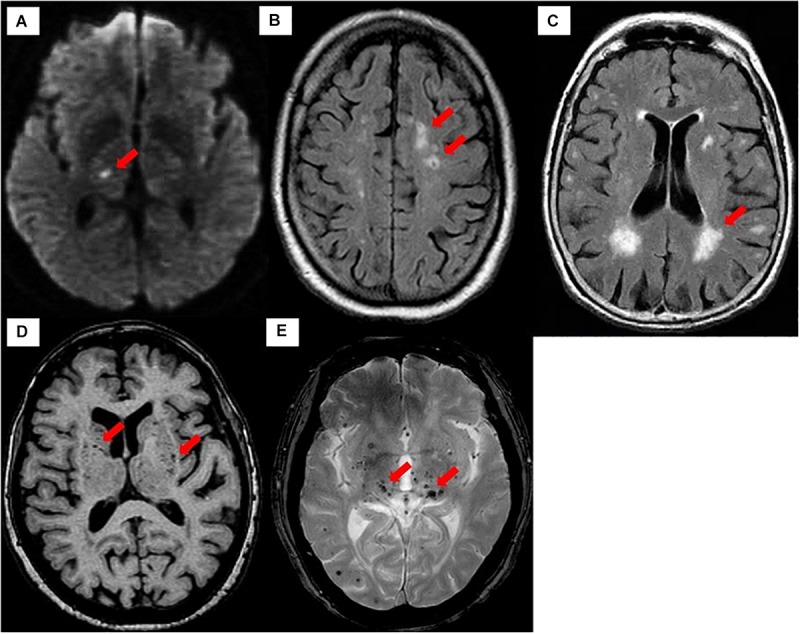
Neuroimaging classification of CSVD based on STRIVE. **(A)** Recent small subcortical infarct on DWI (arrow). **(B)** Lacune on FLAIR (arrow). **(C)** WMHs on FLAIR (arrow). **(D)** Perivascular spaces on T1-weighted imaging (arrow). **(E)** Cerebral microbleeds on T2^∗^-GRE (arrow).

The ischemic consequences of several manifestations of CSVD, such as white matter hyperintensities (WMHs), lacunar strokes, cerebral microbleeds, enlarged perivascular spaces, and small subcortical infarcts, can be detected using magnetic resonance imaging (MRI) (Sorond et al., 2015; Lambert et al., 2015; Yakushiji, 2016; Yakushiji et al., 2018). Wardlaw and colleagues proposed what is known as STRIVE for the methods of visual identification and classification of the CSVD spectrum (Wardlaw et al., 2013b) (Table 2 and Figure 2). The most common imaging spectrum of CSVD is WMHs, which is commonly recognized as small “lacunes” (Latin: for lake) in an aging brain or as bright areas of small non-cavitated high signal intensity on fluid-attenuated inverse recovery (FLAIR) and T2-weighted MRI parameters. The lesion increases with age because it evolves over a few months to years (Ovbiagele and Saver, 2006; Valdés Hernández et al., 2015; Wharton et al., 2015).

**TABLE 2:** STRIVE for the methods of visual identification and classification of the CSVD spectrum (Wardlaw et al., 2013b).

**Neuroimaging marker**	**STRIVE features**	**Aspect in CSVD**	**Comments**
Recent small subcortical infarct	• Recent infarction in one perforating arteriole and its territory • Increased DWI, FLAIR, T2-weighted signal • Decreased T1-weighted signal • Iso-intense T2^∗^-weighted GRE signal	• Infarct can be symptomatic or silent • Number, size, shape and location • Delay from stroke to imaging	• Usual diameter of infarct can be ≤20 mm • Best identified in DWI • Identified symptomatic lesion or imaging features occurred in past weeks hence referred as “recent”
WMHs	• Increase intensity or hyperintensity on T2-weighted, T2^∗^-weighted GRE and FLAIR signal • Iso-intense on DWI and T1-weighted signal • Decrease intensity or hypointense on T1-weighted signal	• Variable location, size, shape, and number	• Mainly located in white matter • Subcortical hyperintensities includes: deep gray matter and brainstem but not include in WMHs • Best identified in FLAIR
Lacune	• Round or ovoid fluid filled cavity mostly in subcortical region • Hyperintensity on T2-weighted signal • Decreased signal in FLAIR and T1-weighted images • Signal similar to CSF • Decreased or iso-intense signal on DWI	• Inclusive of subcortical deep gray matter and brainstem	• Usual diameter around 3–15 mm • Ovoid cavity usually has hyperintense rim • Consistent with previous acute small subcortical infarct or hemorrhage in one perforating arteriole and its territory • Best identified in FLAIR
Perivascular Space	• Fluid-filled spaces that follow the typical course of a vessel as it goes through gray or white matter • Similar signal intensity with CSF • Decrease FLAIR and T1-weighted signal • Increased T2-weighted signal • DWI and T2^∗^-weighted GRE signal seems iso-intense	• Can be found in basal ganglia or centrum semiovale	• Usual diameter around ≤ 2 mm • Mostly linear without hyperintense rim if the image seen parallel to the course of vessel, and rounded if perpendicular
Cerebral Microbleed	• Small, rounded areas of signal void • Iso-intense DWI, FLAIR, T2- and T1-weighted signal • Best seen in T2^∗^-weighted GRE with decreased signal	• Number and distribution are characterized based on lobar, deep and infratentorial (cerebellum ad brainstem)	• Usual diameter around ≤10 mm • Signal void associated with blooming detected on GRE sequence

*CSF, cerebrospinal fluid; CSVD, cerebral small vessel disease; DWI, diffusion-weighted imaging; FLAIR, fluid-attenuated inversion recovery; GRE, gradient recalled echo; STRIVE, Standards for Reporting Vascular changes on neuroimaging; WMHs, white matter hyperintensities.*

In addition, WMHs are also regarded as an ischemic white matter demyelination and can manifest as symptomatic or silent (asymptomatic) brain parenchyma lesions. Interestingly, this so-called “silent” manifestation of CSVD is frequently reported as an incidental finding from brain images of individuals who never experienced any symptom of stroke, which is more frequent among the elderly (Valdés Hernández et al., 2015). It has also been proposed as a prognostic marker following the first symptomatic CSVD presentation, for instance, acute lacunar stroke (Van Norden et al., 2011).

About 95% of asymptomatic manifestations of CSVD are lacunar silent brain infarcts (SBIs) (as seen as WMHs on MRI) and are arguably more prevalent than symptomatic manifestations. The two major contributors to the onset and progression of SBI include age and hypertension (Norrving, 2015). The key differences between SBI and symptomatic lacunar infarcts are their location and size. This is because both SBI and symptomatic lacunar infarcts have similar and overlapping pathological appearances (Bailey et al., 2012). For example, most asymptomatic SBIs are located within the white matter periventricular space (periventricular lesion) and centrum semiovale (deep subcortical lesions) (Kaiser et al., 2014; Wharton et al., 2015), whereas a symptomatic lacunar ischemic stroke affects mostly the sensory and motor tracts (Valdés Hernández et al., 2015).

In addition, healthy white matter is more myelinated than white matter of patients with AD (Bartzokis et al., 2003) and has a higher content of long-chain fatty acids and lower content of water (by 12%) than gray matter. A previous study reported that SBIs are consistently related to age, hypertension, and other cardiovascular risk factors (Prabhakaran et al., 2011). Therefore, individuals with extensive SBIs are at high risk for a future stroke, that is, WMHs serve as a prognostic marker. Nevertheless, it is estimated that SBIs (seen as WMHs) occur in around 30% of healthy subjects over 60 years of age, and with a linear prevalence increment with age (de Leeuw et al., 2001).

Reduced cBF, endothelial dysfunction, oxidative stress, and focal neurological signs are related to cerebral lesions and have been found to correlate with imaging markers, that is, the number and volume of WMHs (Poggesi et al., 2016; Bahrani et al., 2017). Furthermore, WMHs are also associated with cognitive impairment, with the notion that a certain threshold must be achieved before this becomes clinically apparent (Debette and Markus, 2010). Alarmingly, WMHs have also been linked as precursors to developing neuropsychiatric disorders such as schizophrenia (Berlow et al., 2010).

## Experimental Animal Model for CSVD

Investigations using animal models are becoming routine, involving rats and mice or even larger animals such as rabbits and non-human primates, to better understand the development and progression of CSVD. By using animal models, the pathological process of CSVD, such as ischemic white matter lesions following reduced BBB integrity and endothelial dysfunction, can be evaluated. However, as discussed in the previous section of this chapter, CSVD has several different and overlapping pathological features. So the models used ideally should have similar (if not all) CSVD characteristics, that is, diffuse white matter damage, small vessel arteriopathy, and small discrete infarcts, with and/or without cognitive impairment (Edrissi, 2015). Moreover, specific gene expression profiles had been shown in the brain tissues and blood samples from animal models of an ischemic stroke (Jickling and Sharp, 2011). To date, several approaches and animal models have been identified to reflect different aspects of CSVD, and hence mimic the arterial lesion of CSVD and/or brain injury, such as lacunar infarcts and WMHs (Hainsworth and Markus, 2008), as summarized in Table 3. To illustrate this point, this chapter elaborates on the use of rodents as a model for CSVD.

**TABLE 3:** Different approaches used in animal models to reflect certain aspects of CSVD.

**Aspects of CSVD**	**Approaches**
Hypoperfusion/ischemic injury	• BCCAO and stenosis striatal • Endothelin-1 injection • Striatal mitotoxin 3-NPA
Hypertension-based injuries	• SHRSP • Surgical narrowing of the aorta • Genetic mutations, usually in the renin–angiotensin system
Blood vessel damage	• Injected proteases • Endothelium targeting viral infection • Genetic mutations affecting vessel walls

*BCCAO, bilateral common carotid artery occlusion; CSVD, cerebral small vessel disease; NPA, nitropropionic acid; SHRSP, stroke-prone spontaneously hypertensive rats.*

### Hypoperfusion-Based Injuries

The approach used most frequently is bilateral common carotid artery occlusion (BCCAO) or two-vessel occlusion, which reflects the hypoperfusion and/or ischemic injury aspect of CSVD (Choi et al., 2011; Kwon et al., 2015). A previous study has shown that increased white matter degeneration due to hypoperfusion in rats with CCH using the BCCAO approach was also accompanied by an increased loss of oligodendrocytes and neuroinflammation (Choi et al., 2016). Moreover, a recent study supports the fact that a larger number of WMHs (from gadolinium-contrast MRI) found in subcortical brain regions of BCCAO rats is suggestive of the BBB’s perturbed permeability (Arena et al., 2019). Another example of a hypoperfused model is low-density lipoprotein receptor knockout mice to demonstrate the relationship between hypercholesterolemia and CSVD (Hainsworth and Markus, 2008; Tiwari et al., 2018). Besides that, other approaches have also utilized endothelin-1 injection (Capone et al., 2012; Cipolla et al., 2013) and striatal mitotoxin 3-nitropropionic acid (McCracken et al., 2001) to exhibit the impact of hypoperfusion-based injury.

### Hypertension-Based Injuries

Alongside the BCCAO approach, there is another well-established and valid animal model for CSVD – the spontaneously hypertensive stroke prone (SHRSP) rat model (Hainsworth and Markus, 2008; Bailey et al., 2009; Wardlaw et al., 2013c). Selective breeding from the Wistar-Kyoto parent strains produce SHRSP rats, with high arterial blood pressure and incidence of stroke (Yamori and Horie, 1977). This approach reflects the hypertension-based injuries aspect of CSVD. Although the molecular and genetic causes of SHRSP are still elusive, there is evidence of the involvement of renin–angiotensin or the NO signaling system (Bailey et al., 2011b; Hainsworth et al., 2012). The thickening and narrowing of the arterial wall (i.e., in large arteries) are thought to contribute to the hypertensive state in SHRSP rats, and hence the unpredictable cerebral lesions (Baumbach et al., 1988). In another finding, cerebral microangiopathy (due to the BBB breakdown) is present in SHRSP rats using 3-T MRI. Further refinement on detecting microvascular dysfunction related to CSVD in this rat’s model may be achieved by using a higher MRI field strength (i.e., 7T and above) in future research (Mencl et al., 2013). The same group also reported the presence of small but significant perivascular lesions and small vessel thromboses in the histopathological study of the SHRSP rat model (Mencl et al., 2013).

### Blood Vessel Damage–Based Injuries

Another approach is through genetic mutations that affect vessel walls in CSVD. As discussed in the previous section, mutations in the *NOTCH3* gene that encodes transmembrane receptors may contribute to a rare monogenic CSVD such as CADASIL (Chabriat et al., 2009; Joutel, 2011). Previous studies have described the CADASIL-causing R169C point mutation in transgenic mice that carried an artificial chromosome expressing rat *NOTCH3* (Ayata, 2010; Joutel et al., 2010). *NOTCH3* is expressed predominantly in pericytes (Van landewijck et al., 2018); therefore, increased activation of the mutated *NOTCH3* gene is linked with a reduced pericyte function (i.e., due to platelet-derived growth factor receptor-signaling β dysregulation) that contributed to the arteriovenous malformations and white matter lesions as precursors of CADASIL (Kofler et al., 2015; Montagne et al., 2018).

### Animal Models’ Merit to Understand CSVD in Humans

To date, studies on animal models that can replicate human CSVD are still limited (Bailey et al., 2011a; Hainsworth et al., 2012). The main reason behind this is the fact that most experimental animal studies are limited to mice and rats. Compared to rats and mice, humans have a longer lifespan, a larger brain size, bigger vessel dimensions, and a higher gray to white matter ratio. Although mice capillaries do resemble those of humans, rodent arteries have little resemblance to humans’ deep penetrating arteries in the subcortical region that are frequently implicated in CSVD (Giwa et al., 2012). That said, a recent study in mice with single penetrating arteriole occlusions showed that a local collapse of microvascular function contributes to tissue damage, which mimics the pathophysiology induced by microinfarcts found in the human brain (Taylor et al., 2015). Moreover, the mimicry of animal models in human CSVD includes diffuse damage to any deep white matter structures, including rarefaction, vacuolization, or other damage to the myelin, or damage to the axonal tracts. Besides, several models with specific features that resemble human CSVD are summarized in Table 4.

**TABLE 4:** Animal model, features, and CSVD correlates (Lee et al., 2007; Jiwa et al., 2010; Joutel et al., 2010; Schreiber et al., 2013; Silasi et al., 2015).

**Animal models**	**Features**	**CSVD correlates**
Stroke-Prone Spontaneously Hypertensive Rats (SHRSP)	• To elucidate early histological changes in CSVD	• Displayed local BBB breakdown that was determined with MRI • Endothelial injuries lead to multiple sites with BBB leakage which cause damage to the vessel wall and results in vessel ruptures and microbleeds
*NOTCH3* transgenic mice	• Mimic CADASIL • Resembles age-related sporadic CSVD	• *NOTCH3-*R169C mice develop diffuse white matter lesion in corpus callosum, internal capsule and striatal white matter bundles, no change in BBB function was detected
BCCAO in rats	• Mimic bilateral common carotid occlusion • Model for the study of vascular cognitive impairment	• White matter lesions characterized by vacuolation of myelin, axonal damage, and demyelination in corpus callosum, internal capsule, and caudate putamen • BCCAO in rat mimics chronic hypoperfusion (i.e., chronic cerebral hypoperfusion, CCH rats) of CSVD and arteriosclerosis
Thy1-GFP transgenic mice	• Visualized the impact of micro-occlusions on neuronal structure	• Occlusions in CSVD are produced through endovascular injection of fluorescent microspheres • Micro-occlusions in the hippocampus produce cell loss or neuronal atrophy • Disruption of axons in white matter tract, striatum and thalamus

*BBB, blood–brain barrier; BCCAO, bilateral common carotid arteries occlusion; CADASIL, cerebral autosomal dominant arteriopathy with subcortical infarcts; CCH, chronic cerebral hypoperfusion; CSVD, cerebral small vessel disease; GFP, green fluorescent protein.*

Therefore, the use of an animal model to study the natural history of CSVD can help us explore the pathomechanism of CSVD up to the cellular and molecular levels. Besides, animal models also provide a potential benefit of testing the effects of developed drugs or other interventions on the pathomechanism of CSVD. Finally, experimental animal models may provide a way for us to examine the interactions of multiple pathomechanisms of CSVD, for example, the interactions of CSVD with its comorbidities such as obesity, diabetes mellitus, and AD, which are clinically relevant. Further details on the involvement of animal models in understanding the systems biology of CSVD, potential biomarkers, and current and future therapeutic approaches for CSVD are discussed in next section of this chapter.

## Relevant Aspects on Systems Biology

**FIGURE 3:**
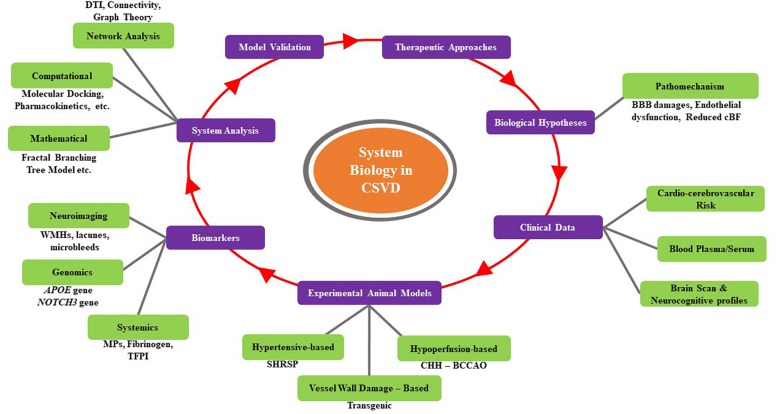
Summary of a general workflow of systems biology for CSVD. This figure summarizes the overall approach, highlighting some of the options available at each step.

Cerebral small vessel disease has a crucial role in lacunar stroke even in brain hemorrhages and is one of the leading causes of cognitive decline and functional loss in elderly patients (Pantoni, 2010). Interactions between genetic, cellular/molecular, and environmental factors (aging and vascular risk-hypertension) influence the development and progression of CSVD (Cai et al., 2015; Verhaaren et al., 2015). Genetic and cellular/molecular factors play a key role in terms of unraveling the pathomechanism of CSVD (Verhaaren et al., 2015). Hence, applications in systems biology in terms of exploring and identifying genetic and cellular/molecular architectural mechanisms with the advances in technological approaches (i.e., computational, mathematical, and network analyses) may provide a greater understanding of CSVD and ultimately possibly lead to the development of novel preventive and therapeutic measures (Ehret et al., 2011; Giese and Rost, 2017). Figure 3 summarizes a general workflow of systems biology in CSVD. This figure summarizes the overall approach, highlighting some of the options available at each step.

As discussed, some but not all animal models exhibit clinicopathological features that resemble human CSVD. The exploration and identification of cellular and molecular architectural mechanisms and genetic testing using animal models have been proven to represent human CSVD (Hainsworth and Markus, 2008). For example, several lines of evidence associating CSVD with the increased permeability of the BBB and endothelial dysfunction have been found (Cuadrado-Godia et al., 2018). Apart from that, endothelial dysfunction and reduced BBB integrity were found to be associated with the severity of cerebral white matter lesions following a significant decrease in EC integrity in diseased white matter compared with that in normal white matter (Young et al., 2008). Besides, the animal model also can exhibit any CSVD-like vessel pathology that includes reduced BBB integrity and changes in small vessel walls (Hainsworth and Markus, 2008).

Several categories of animal models that have been used to understand CSVD include hypoperfusion/ischemic injury, hypertension-based models, vessel damage, mutations and vessel damage, and interventions (see Table 4). A majority of these models are used to study the target mechanism of BBB damage, endothelial dysfunction, reduced cBF, and the involvement of genetic counterparts; these appeared to be suitable animal models for research in understanding CSVD (Kraft et al., 2017). Further details on specific cellular and molecular mechanisms with genetic contributions to CSVD will be discussed in this section.

### Pathomechanism of BBB Damage in Relation to CSVD

**FIGURE 4:**
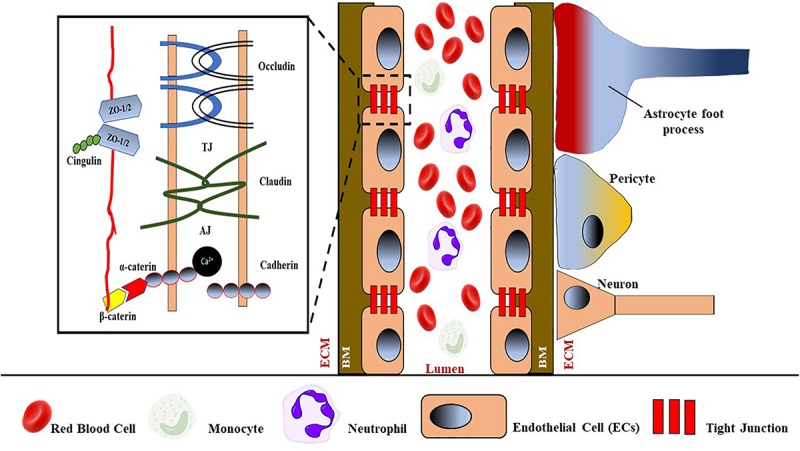
Schematic representation of BBB with its components such as tight junctions (TJs), endothelial cells (ECs) that attach to the basement membrane (BM), astrocytes foot process, pericytes, neurons that are separated from ECs by the extracellular matrix (ECM). TJs consist of occluding, claudin, and adhesion junctions such as cadherin. ZO-1/2, zona occluding 1 or 2.

The physical and functional integrity and health of the brain are basically maintained by the BBB, which is specialized to prevent pathogens and circulating immune cells from entering the vulnerable system of the brain and causing damage. Generally, the BBB consists of monolayer ECs that are connected by defensive tight junctions, hence preventing extracellular molecules from passively entering the brain (Wolburg and Lippoldt, 2002) (see Figure 4). Apart from that, the existing interactions of blood components, neutrophils and monocytes, in blood circulation with the luminal surfaces of ECs, are also considered as part of the BBB and play an important role in immune surveillance. Moreover, three transmembrane proteins collectively form a tight junction between ECs: occludins, claudins, and cadherins. The interactions between these proteins serve as a protective gate from the passive leakage of extracellular molecules.

**FIGURE 5:**
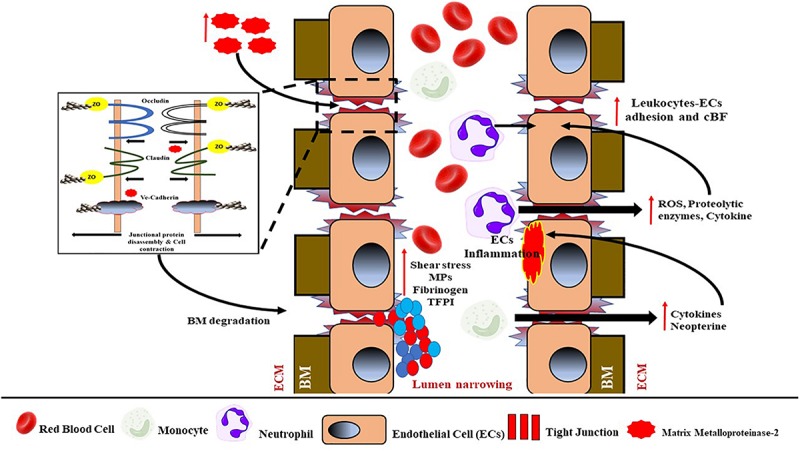
Schematic representation of the mechanism of BBB damage and endothelial dysfunction. Increased activity of matrix metalloproteinase-2 (MPP2) from ECM will cause tight junctions (TJs) to dissemble. TJ damage will eventually lead to basement membrane (BM) degradation and endothelial damage, and hence lead to endothelial dysfunction. BBB damage will permit the infiltration of neutrophils, monocytes, and blood components into the ECM. Activated neutrophils induce the activation of ROS, proteolytic enzymes, and cytokines, thus causing higher leukocyte–ECs adhesion and reduced cBF. Meanwhile, activated monocytes will be induced by cytokine and neopterin to cause inflammation in the ECs. Increased shear and oxidative stress from the system also will cause the activation of blood components and increased production of MPs, reduced TFPI, and increased fibrinogen accumulation, finally causing lumen narrowing to further reduce cBF. ROS, reactive oxygen species; cBF, cerebral blood flow; MPs, microparticles; TFPI, tissue factor pathway inhibitor.

Damage to the integrity of the BBB can provide entry to extracellular molecules, including immune cells and invasive pathogens, thus interrupting brain function (Hawkins and Davis, 2005). Generally, damage of the BBB, that is, increased BBB permeability, is due to the disassembly of tight junction proteins; and the progressive BBB damage and increased permeability may eventually cause EC basement membrane degradation and ECM material accumulation, thus influencing vessel wall stiffening. The subsequent increased infiltration of immune cells and inflammation are also a result of increased BBB permeability. On the other hand, the secretion of cytokines and neopterin by activated monocytes/macrophages can cause inflammation of ECs by disrupting their ECM, resulting in the BBB breakdown. The BBB disruption can also be caused by matrix metalloproteinase 2 (MPP2), which is an ECM degradation enzyme known to degrade the tight junction proteins in rodents (Nakaji et al., 2006). When the ECM of the BBB is damaged, it will eventually lead to increased BBB permeability and the penetration of immune cells, followed by inflammation. Moreover, disruption of the BBB due to deposition of blood and platelet components, that is, fibrinogen and microparticles, can also worsen the BBB damage (see Figure 5).

Several studies have suggested that changes in walls of small vessels in the brain (i.e., due to BBB disruption) may lead to ischemic damage, causing WMHs, lacunae and microbleeds associated with CSVD (Cai et al., 2015; Wardlaw et al., 2017; Zhang et al., 2017). Hence, this further supports the fact that the disturbance of BBB integrity can cause changes in walls of small vessels in the brain (Kraft et al., 2017). Recently, various *in silico* methods and models in the pathology of the BBB, that is, BBB computational pathology using mathematical approaches, have been used to study and predict BBB integrity up to the molecular level, and its relationship with cerebral damage (Shityakov and Förster, 2018). The majority of the computational approaches incorporate molecular dynamics (MD), molecular docking simulations, pharmacokinetics, and finite element methods, but lack details on the pathomechanism of BBB damage (Shityakov and Förster, 2014; Shityakov et al., 2015; Del Razo et al., 2016).

Although computational approaches provide limited details, several types of *in silico* computational approaches are used to study BBB-related pathology, hence lending support to the involvement of the BBB in CSVD. The first approach is MD, a method used at the molecular level of complexity, for example, a model to study the BBB, mutated or misfolded proteins and transporters, and small molecule permeation across the BBB. The MD approach enables the investigation of impaired protein structure and function and drug-like molecule cytotoxicity at the BBB. Besides MD, finite element methods and pharmacokinetics target the organ (i.e., the brain) as the level of complexity, opening a new window into investigations on cerebral injury, that is, BBB leakage and permeation (Shityakov and Förster, 2018).

In a similar development, efforts have been made to model the circulatory system using a mathematical model (Šutalo et al., 2014; Takahashi, 2014). Fractal geometry is a mathematical model used frequently to study the complexity of patterns and processes in a wide range of natural phenomena observed in several fields, such as medicine (Di Ieva et al., 2014), geology (Nikora and Sapozhnikov, 1993), and cosmology (Dickau, 2009). The term “fractal” was first coined by Benoit B. Mandelbrot in 1975 and is derived from the Latin adjective “fractus,” meaning “broken” or “fractured” (Di Ieva, 2016). Fractal concepts are used to describe irregular natural structures such as blood vessels (Weibel, 2005) and rivers (Nikora and Sapozhnikov, 1993). These structures appear self-similar under various degrees of scale, exhibit scaling properties, and possess non-integer (fractional) dimensions (Di Ieva et al., 2014; John et al., 2015). For instance, the human circulatory system is composed of a complex network of branching blood vessels in which vessels of smaller caliber represent a repetition of larger blood vessels on a smaller scale (Weibel, 2005). A recent study discovered that the fractal approach could be used to model blood flow through the cerebral vasculature (Šutalo et al., 2014). In effect, the fractal approach might provide us a better understanding of the disease of interest. Notably, the fractal approach has not yet been explored extensively in CSVD, and warrants further research.

### Endothelial Dysfunction and Nitric Oxide Signaling in Relation to CSVD

Endothelial dysfunction can lead to CSVD by various mechanisms that can cause hypoperfusion or reduced cBF. cBF is regulated mainly by NO signaling, which has been identified as a marker for endothelial dysfunction (Deplanque et al., 2013). As discussed, endothelial dysfunction can increase BBB permeability and subsequently lead to brain parenchyma lesions. Endothelial dysfunction can alter the secretion of the releasing factor from the ECs, which affects oligodendrocyte survival and leads them to apoptosis. Signaling between dysfunctional ECs and oligodendrocytes may alter their ability to survive up to the damage caused by hypoperfusion in humans with CSVD. Moreover, endothelial dysfunction can impair the movement of OPCs upon blood vessels and thus lessen the repair process (Rajani et al., 2018). In CSVD, the vessel lumen restriction is thought to lead to a state of chronic hypoperfusion of the white matter, eventually resulting in the degeneration of myelinated fibers because of repeated selective oligodendrocyte death.

This ischemic mechanism due to endothelial dysfunction has been demonstrated in experimental animal models, for example, over-expression of the *p53* gene could mediate oligodendrocyte apoptosis, thus resulting in demyelination in two ways: by enhancing endoplasmic reticulum–mitochondria interactions and by triggering the activation of the *E2F1* gene–mediated apoptosis pathway (Pantoni, 2010; Ma et al., 2017). On the other hand, endothelial dysfunction can also be mediated by the activation of neutrophils, lymphocytes, monocytes, and platelets that can act on ECs to either weaken or strengthen the barrier. The aforementioned mediators exert their effects on barrier function by altering the width of the intercellular junctions, or through changes in junctional proteins and/or the EC cytoskeleton. For example, activated neutrophil release can impair endothelial barrier function, following the activation of reactive oxygen species (ROS), proteolytic enzymes, and cytokines. Products of neutrophil activation can alter barrier function by acting on the EC cytoskeleton, junctional proteins, and the endothelial glycocalyx. ECs exposed to ROS exhibit an increased permeability response that has been linked to disruption of the inter-endothelial junction, actomyosin contraction, gap formation, and an altered expression and phosphorylation state of junctional adhesion molecules (van Wetering et al., 2002; Monaghan-Benson and Burridge, 2009; Rodrigues and Granger, 2015).

NO can be a positive or negative modulator that may affect the endothelial barrier function. The protective role of NO lies in its ability to inhibit leukocyte–EC adhesion. NO-synthase inhibition increases the permeability of EC monolayers, a response associated with the formation of stress fibers and the disruption of adherens junctions (Kubes and Granger, 1992; Rodrigues and Granger, 2015). NO interacts with the connexin of tight junctions and enzyme NO signaling in ECs, and the alteration in this interaction can influence the vascular disease condition (Looft-Wilson et al., 2012). Moreover, the investigation revealed a significant reduction in NO and L-citrulline concentrations and a rise in L-arginine, and the precursor of these substances, in the patients’ jugular blood. This can be the result of endothelial dysfunction and deficient synthetase expression (Neri et al., 2006). NO can also modulate Rho kinase activity in cerebral microvessels, such that the inhibition of NO synthase activity increases the influence of Rho kinase on the vascular tone (Didion et al., 2005). The loss of NO during a disease can start a vicious cycle: increased Rho kinase activity leading to a decrease in NO synthase-derived NO, which may further increase Rho kinase activity (De Silva and Miller, 2016).

### Genetic Factors in CSVD

Progressive arteriopathy, white matter disease, subcortical infarcts, and clinical manifestations in stroke and dementia are several shared features between sporadic CSVD and monogenic CSVD. Moreover, the heritability of CSVD ranges between 55 and 73%, which is significantly higher than that of carotid atherosclerotic CSVD (Carmelli et al., 1998; Atwood et al., 2004). Therefore, it is now clear that hereditary CSVD is genetically heterogeneous and represents different disease entities (Federico et al., 2012) (Table 5). The identification of underlying genes involved in the disease stratification is now possible with advances in DNA sequencing technology as well as genetic linkage analyses. For example, studies on the involvement of genes associated with cerebral white matter lesions give better insights into the pathomechanism of CSVD (Woo et al., 2014; Verhaaren et al., 2015; Lopez et al., 2015). In this section, we briefly describe the genetics of the most frequent CSVD and their pathomechanism.

**TABLE 5:** Different entities of heterogenous hereditary CSVD (Joutel et al., 2010; Taguchi et al., 2013; Beaufort et al., 2014; Haffner et al., 2016).

**Type**	**Mendelian inheritance**	**Gene (s)**	**Pathological features**	**Animal models**	**Remarks**
CADASIL	Autosomal dominant	*NOTCH3*	• Pure SVD • NOTCH3 deposits (granular osmiophilic material, GOM) • Loss of vSMC • Parenchymal arterioles and capillaries walls thickening	*TgNOTCH3*^*R*169*C*^	• Commonly followed by stroke and dementia • Other clinical manifestation such as psychiatric disturbance and migraine with aura
CARASIL	Autosomal recessive	*HTRA1*	• Loss of vSMC • Reduction of extracellular matrix mural • Splitting of internal elastic lamina • Thickening of intimal layer and luminal stenosis	*HtrA1^–/–^*	• Commonly followed by stroke and dementia
RVCL	Autosomal dominant	*TREX1*	• Defect of capillaries basement membrane • Luminal stenosis • Vascular necrosis • Adventitial fibrosis	–	• Rarely involve stroke but occasionally followed by dementia • Other clinical manifestation includes retinopathy, cognitive impairment, psychiatric disturbance and migraine
Fabry’s disease	X-linked	*GLA*	• Multifocal leukoencephalopathy • Loss of small myelinated and unmyelinated fibers	*TgG3S/GLA^–^*^/^*^–^*	• SVD with additional involvement of large arteries • Commonly followed by stroke and occasionally with dementia • Other clinical manifestation includes cataract, renal failure, and neuropathic pain

*CADASIL, cerebral autosomal dominant arteriopathy with subcortical infarcts and leukoencephalopathy; CARASIL, cerebral autosomal recessive arteriopathy with subcortical infarcts and leukoencephalopathy; CSVD, cerebral small vessel disease; GLA, galactosidase-A; HTRA1, high-temperature requirement protein A1; RVCL, retinal vasculopathy with cerebral leukodystrophy; SVD, small vessel disease; vSMC, vascular smooth muscle cell.*

Multiple factors and pathomechanisms that lead to vascular and parenchymal injury in CSVD have been reported previously through mechanistic studies on experimental animal models and even humans (Wardlaw et al., 2013c; Haffner et al., 2016). With certain considerable overlapping pathological features of sporadic and monogenic CSVD, however, some features, such as BBB breakdown, has not been consistently demonstrated in monogenic CSVD. However, through genetic and mechanistic studies, the involvement of the ECM in monogenic CSVD has currently emerged as a novel aspect of the disease pathomechanism. Moreover, the ECM is now thought to be a key player in multiple forms of CSVD (Haffner et al., 2016).

#### Notch3 Signaling in CADASIL

Experimental animal models (i.e., mouse) have demonstrated that the maturation of vascular smooth muscle cells (vSMCs) and arterial differentiation depend on the activity of cell signaling that involves the Notch family of cell signaling receptors, especially Notch3 (Domenga et al., 2004). The stereotype nature of CADASIL mutations supports the notion of a toxic gain-of-function mechanism due to *NOTCH3* gene aberrations. Previous studies have shown that CADASIL with mutant *NOTCH3* aggregates accumulates in the ECM of small arteries, arterioles, and capillaries (Joutel et al., 2000, 2001; Monet-Lepretre et al., 2013).

**FIGURE 6:**
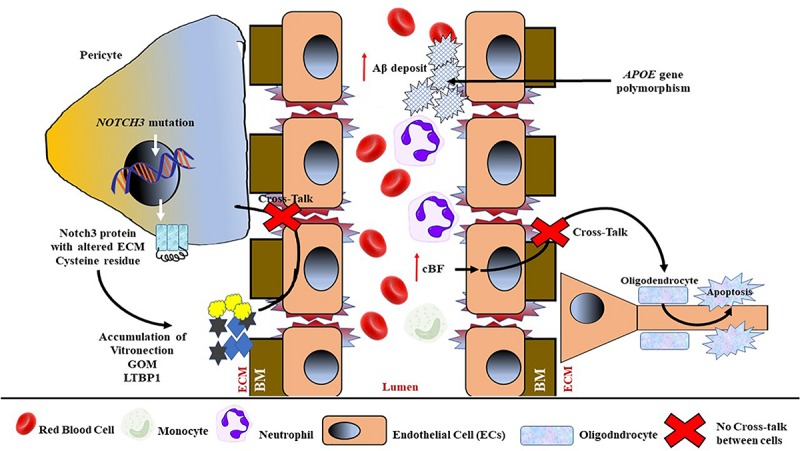
Schematic representation of genetic contribution toward the mechanism of BBB damage and endothelial dysfunction, especially in hereditary CSVD. Mutations of the NOTCH3 gene in pericytes result in a mutated Notch3 protein, with an altered cysteine residue at the ECM to be multimerized by recruiting matric proteins, that is, vitronectin, granular osmiophilic material (GOM), and tissue inhibitor of metalloproteinases-3, and latent transforming growth factor β (TGF-β)-binding protein-1 (LTBP1). The accumulated multimer will cause further damage to ECs and BM, and hence interfere with the cross-talk between pericytes and ECs. Reduced cBF also interferes in the cross-talk between ECs and neuronal oligodendrocytes, thus inducing oligodendrocyte apoptosis. The accumulation of amyloid β (Aβ) at the luminal wall due to apolipoprotein E (APOE) polymorphism will further induce BM degradation and EC dysfunction and luminal narrowing.

Although the exact pathomechanism involving mutant Notch3 aggregation still needs further investigation, previous studies have proposed that the co-aggregation of mutant Notch3 and other proteins suggest that additional proteins are recruited into Notch3 deposits (Duering et al., 2011; Kast et al., 2014). For example, once the mutated Notch3 related to CADASIL is secreted and participates in cell signaling, it multimerizes and initiates the formation of large aggregates and deposits that include matrix proteins, such as vitronectin, tissue inhibitor of metalloproteinases-3, and latent transforming growth factor-β (TGF-β) binding protein-1 (LTBP1); instead of undergoing clearance from the ECM, these aggregates alter the normal physiological function of blood vessels and their surroundings (see Figure 6).

As mentioned in Section “Dynamic Pathological Processes of CSVD,” the stabilization of the BBB involves not only ECs but also pericytes and OPCs. Pericytes are known to play a role in the maturation and maintenance of BBB integrity; abnormal cross-talk between pericytes and ECs due to *NOTCH3* gene mutations can also cause damage in the BBB (see Figure 6). Animal studies have shown that pericytes also play a significant role in CSVD, as emphasized in CADASIL (Armulik et al., 2005; Ihara and Yamamoto, 2016; Cuadrado-Godia et al., 2018). Moreover, mutations in the *NOTCH3* gene alter the number of cysteine residues in the extracellular domain of the Notch3 protein, which eventually lead to the extracellular accumulation of granular osmiophilic material. *NOTCH3* is also expressed in pericytes and mutations in this gene revealed a loss of ECs and pericytes that disrupted the BBB (Coupland et al., 2018).

#### TGF-β Signaling in CARASIL

The observations conducted on experimental animal models and patients with cerebral autosomal recessive arteriopathy with subcortical infarct and leukoencephalopathy (CARASIL) up to the cellular level had suggested that TGF-β signaling pathways were significantly involved in the pathomechanism of monogenic CSVD. Due to its vast expression, TGF-β has multiple biological functions including a regulatory role in vascular development (ten Dijke and Arthur, 2007). The TGF-β signaling pathway is thought to have a link with high-temperature requirement protein A1 (HtrA1), which is expressed in multiple TGF-β-relevant tissues (Hara et al., 2009) and HtrA1 has been suggested to inhibit TGF-β in various experimental models (Oka et al., 2004; Launay et al., 2008; Zhang et al., 2012; Graham et al., 2013).

Mutations of the *HTRA1* gene may lead to fragility of the vascular wall in CARASIL. The *HTRA1* gene encodes an evolutionarily conserved serine protease. Mutations in CARASIL generally due to loss of HtrA1 activity that impaired substrate processing, hence, serve as primary disease mechanism. HtrA1 is primarily located and functions in the ECM; hence, it helps to degrade certain substrates located in extracellular compartments (Beaufort et al., 2014). However, aberrations and mutations in the *HTRA1* gene can lead to abnormally aggregated elastin, the main protein component of the elastic lamina, presumably being associated with its fragility (Ito et al., 2018). For example, during the secretion, the interaction of LTBP-1 with fibronectin (a matrix protein) helps facilitate the latent TGF-β to be incorporated into the ECM. Following this, the clearance of mature TGF-β from the ECM is generally facilitated by the proteases HtrA1 through LTBP-1 cleavage. Therefore, *HTRA1* gene mutations can result in loss of HtrA1 activity, thus interfering in TGF-β in CARASIL and subsequently leading to vSMC degeneration (Nozaki et al., 2014).

The HtrA1 mutant in the mouse brain model has been reported to markedly reduce protease activity. Protease activity and *HTRA1* gene mutations differ according to their locus, and these differences might correlate with the severity of the vascular changes and leukoencephalopathy (Nozaki et al., 2016). TGF-β signaling was found to be associated with downregulated genes in the basal ganglia of patients with CSVD, suggesting an absence of TGF-β-induced detrimental effects on vSMCs in this area (Ritz et al., 2017). In addition to the contribution of genetic mutations in monogenic CSVD, several genetic variants are also related to sporadic CSVD, that is, genetic mutations in amyloid CSVD and the involvement of oxidative phosphorylation gene mutations in CSVD related to lacunar infarcts.

### Signaling Pathway in Amyloidogenic CSVD

#### Apolipoprotein E (APOE)

Amyloid-β (Aβ) protein accumulation in cerebral capillaries has been widely studied and shown to affect BBB integrity, which leads to a loss of tight junction proteins and thus to increased BBB permeability. The progressive deposition of Aβ in the walls of cortical and leptomeningeal small arteries can lead to vessel dysfunction and brain parenchymal injury, thus causing vascular occlusion and rupture (see Figure 6). On the other hand, *APOE* gene polymorphisms have been associated with amyloidogenic CSVD and serve as the strongest genetic factor for the disease including AD. Two main types of *APOE* genes are involved in CVSD: *APOE* ε4 and *APOE* ε2, and their roles have been summarized in Table 1.

These genes are involved particularly in the amyloidogenic pathway, whereby polymorphisms of these genes in mice have been shown to cause the loss of pericytes and EC cross-talk, and are thus associated with BBB disruption (Schuur et al., 2011). Moreover, the presence of two *APOE* ε4 alleles was related with the presence of lacunae. Additionally, this finding suggested that there is a crucial involvement of Aβ clearance in the pathogenesis of lacunae. Moreover, it is widely accepted that APOE is associated with aging; hence, it serves as a risk factor that influences CSVD (Liu et al., 2010; Schuur et al., 2011).

#### Single-Nucleotide Polymorphisms (SNPs) and Oxidative Phosphorylation

SNPs in the gene for SORL1, a low-density lipoprotein receptor class, has been shown to be associated with CSVD. Deficiency of *SORL1* gene expression leads to an increased Aβ level and enhanced amyloid pathology in the brain (Rogaeva et al., 2007). For example, a microbleed occurred in the immediate perivascular region from amyloid angiopathy (Schuur et al., 2011; McCarron and Smith, 2017; MacGregor Sharp et al., 2018). Thus, there is an association between reduced *SORL1* gene expression and a microbleed, which is suggested by the role of Aβ in neurodegeneration through the perivascular region (Weller et al., 2008). The *SORL1* gene may also be linked to its role in a microbleed, which is one of the phenotypes of CSVD (Cohn-Hokke et al., 2017; Maple-Grødem et al., 2018).

Focusing on aggregate measures of genetic variation rather than individual SNPs, a previous study had identified several variants within a larger set of oxidative phosphorylation genes collectively associated with an increased risk of lacunar stroke (Anderson et al., 2013). The oxidative phosphorylation genes are encoded by mitochondrial and nuclear DNA. Lacunar stroke showed associations with genetic risk scores in oxidative phosphorylation as a whole, complex I, and complex IV. These findings are complemented by another study that found a genetic score of mitochondrial variants to be associated with WMH volume in patients with ischemic stroke (Anderson et al., 2011). Apart from that, aggregated Aβ protein can reduce mitochondrial respiration in neurons and induce ROS production, which lead to the dysfunction of mitochondria (Canevari et al., 1999; Casley et al., 2002). A previous study also shows that the overproduction of mitochondrial ROS in vascular diseases caused the dysfunction of mitochondria, hence contributing to the progression of CSVD induced by hypercholesterolemia (Angelova and Abramov, 2018). Collectively, these findings suggest that genetic variations in oxidative phosphorylation influence small vessel pathobiology, although the exact mechanisms remain to be determined.

### Other Genetic Variants Related to CSVD

As discussed, CSVD exhibits perturbed end-artery function and has an increased risk for stroke and age-related cognitive decline. The increment in BBB permeability plays a crucial role in the disease onset and progression. However, there is also some involvement of genetic material, whereby their aberration may interfere with BBB integrity and disease stratification. For example, BBB integrity is maintained via proteins in the matrisome, which involves the interaction between multiple genes including the forkhead transcription factor (*FOXC1*) gene, and damage of the BBB due to aberrations and mutations of the *FOXC1* gene may permit the entry of pathogens or immune cells and disrupt brain function, thus leading to the onset and progression of CSVD (Chauhan et al., 2016).

Moreover, the alteration of the *FOXC1* gene has been associated with the presence of extensive WMHs, whereby the inhibition of the *FOXC1* gene may disturb the signaling of platelet-derived growth factor, causing impaired neural crest migration and the recruitment of mural cells, which are essential for vascular stability. In addition, there is also a link between FOXC1-interacting transcription factor (*PITX2*) and CSVD, and both patients with *PITX2* gene mutations and murine Pitx2^–/–^ mutants displayed brain vascular phenotypes. Together, these results extend the genetic etiology of stroke and demonstrate an increasing developmental basis for human cerebrovascular disease (French et al., 2014; Chauhan et al., 2016; Tan et al., 2017).

## The Opportunity of EarLy Disease Biomarkers

The National Institutes of Health Definitions Working Group defined a biomarker as a characteristic that is objectively measured and evaluated as an indicator of normal biological processes, pathogenic processes, or pharmacological responses to intervention (Biomarkers Definition Working Group, 2001). The animal models of CSVD can be used to expedite the optimization of imaging markers for clinical use and their pathology develops in a shorter time frame than in humans, enabling a high throughput of biomarker testing. Genetic mutations in transgenic mice can produce different CSVD-like pathophysiological features in isolation. Biomarkers for neurodegenerative disorders are essential to facilitate disease diagnosis, ideally at early stages, monitor disease progression, and assess responses to existing and future treatments.

### Biomarker 1: Neuroimaging-Based Biomarkers

Radiological features (see Table 2) are the primary clinical biomarkers of CSVD that can be visualized routinely on computed tomography and MRI. Small vessels cannot be visualized through *in vivo* and their pathological evidence is very limited. Therefore, neuroimaging is accepted as a necessary method in diagnostic markers and research of CSVD (Banerjee et al., 2016; Chen et al., 2018). Several imaging modalities have been developed and implemented in animal models to image molecular and cellular processes *in vivo*. Imaging modalities might be categorized into two groups: those providing mainly structural information, such as computed tomography, MRI, or ultrasound; and those aiming mainly at functional or molecular information, like positron emission tomography (PET), single photon emission computed tomography (SPECT), or optical imaging (Waerzeggers et al., 2010).

Compared with PET and MRI, optical imaging techniques are most cost-effective and time-efficient, require less resources and space, and have excellent temporal resolution. Nevertheless, the disadvantages of these techniques are the limited spatial resolution and depth penetration, hence are only suitable for small animal research because of the lack of optimal quantitative or tomographic information. PET and SPECT as a nuclear imaging technique have a high sensitivity, with which a specific tracer accumulation with very low levels can be detected, but have an inherently limited spatial resolution. MRI is the most widely used technique that has a spectacular spatial resolution unlimited by detector geometry, as with nuclear imaging, or by tissue scattering properties, as by optical imaging; however, its temporal resolution is limited, and molecular probe detection is several orders of magnitude less sensitive than nuclear imaging techniques.

A multitude of animal models have been established to mimic human disorders, ranging from interventional models (such as xenograft, neurotoxic, or mechanical lesion models) to knockout and transgenic (mono-, bi-, or trigenic through cross-breeding) animals. With the development of these animal models, non-invasive techniques to assess functional, biochemical, and anatomical disease-related changes have become indispensable, and a variety of small animal models have been developed for the imaging scanners with high sensitivity, specification, and resolution. Furthermore, the findings in mouse models obtained by small animal PET/SPECT and MRI scanners can be compared directly to the human situation with clinical scanners and represent true translational research from bench-to-bedside and back to the bench again.

On the other hand, cerebral perfusion, cerebrovascular reactivity, BBB permeability, and white matter microarchitecture are accessible through MRI; these pathologies are altered in CSVD. Cerebrovascular reactivity may occur at early stages of CSVD and is correlated with future development of WMHs, while reduced cBF may predict the future risk of dementia. Newer approaches, such as diffusion tensor imaging (DTI) (e.g., graph theory–based analysis of network of DTI connectivity between cortical nodes and analysis of histogram of mean diffusivity of cerebral white matter), had received more attention for the assessment of CSVD (Smith and Beaudin, 2017). DTI is used widely in aging and neurodegenerative studies and can also be used as a potential surrogate biomarker in disease onset and progression, especially in an animal model. The utility of DTI as a tool to interpret the order in which white matter disease and neurodegeneration occur is challenging due to the difficulty in interpreting DTI quantitative parameters, conflicting results between studies, and the possibility of combined effects from multiple causes such as ischemic mechanisms, cerebrovascular disease, and reactive gliosis (Huang et al., 2007).

Diffusion tensor imaging studies in mouse models that exhibit specific types of white matter abnormalities may become a guide for information on the root cause of signal changes. One study in the shiverer (shi) mouse model of dys-myelination and demyelination using cuprizone treatment suggests that reductions in the myelination of the corpus callosum increase radial diffusivity (i.e., increase demyelination) (Song et al., 2005). In more subtle white matter pathology induced by hypoperfusion, mice exhibited a lower fractional anisotropy in the corpus callosum that correlated with measures of reduced myelin integrity (Holland et al., 2011). In some rodent models of axonal degeneration and injury, reduced axial diffusivity has been observed in affected white matter regions (Song et al., 2002; Zhang et al., 2009). Mouse models can be used to better understand the relationship between DTI parameters and disease. The careful breeding and housing of transgenic mouse models of CSVD may reduce group variability by controlling the external factors, such as exercise and diet, that will be affected in human studies. Histological analysis can be used to identify the physical tissue changes driving diffusion measurements and to draw correlations between DTI indices and the presence of known pathologies in CSVD. The expedient progression of pathology in models allows rapid longitudinal studies of DTI measurements in early and late stages of the disease (Vincze et al., 2008).

In summary, the new and advanced quantitative neuroimaging techniques are not ready for routine radiological practice, but are already being employed as monitoring biomarkers in the newest generation of trials for CSVD (Smith and Beaudin, 2017).

### Biomarker 2: Amyloid Pathology

CAA is a common amyloidal form of CSVD, and its incidence is mostly related to advanced age (Biffi and Greenberg, 2011). Based on the specific location of amyloid deposition and allelic difference, two pathological subtypes of CAA have been recognized: CAA type 1, characterized by amyloid in cortical capillaries; and CAA type 2, in which amyloid deposits are restricted to leptomeningeal and cortical arteries, but not capillaries (Table 1) (Thal et al., 2002). Predominantly, CSVD is characterized by endothelial damage, BBB breakdown, and subsequent small vessel wall degeneration, even though CAA is characterized mainly by the deposition of Aβ in the basement membranes of capillaries and smaller arteries (Charidimou et al., 2012; Wardlaw et al., 2013c).

A study on SHRSP rats, as a valid model of non-amyloid CSVD, found the mutual occurrence of non-amyloid CSVD and CAA, as is commonly found in the aging brain (Held et al., 2017). Spontaneous CAA development in a non-transgenic, non-amyloid CSVD model suggests that there should be some mechanisms connecting the two small vessel disease entities. In the aging brain, non-amyloid CSVD and CAA can be considered as part of the same vascular disease spectrum. Therefore, these SVD entities could be interrelated through Aβ transport disturbances and ECM protein alterations. The overlap among non-amyloid CSVD and CAA could result in similar treatment concepts perceptively.

In addition, recent evidence suggested that through the use of a novel cerebrospinal fluid biomarker of BBB-related soluble platelet-derived growth factor receptor-β and capillary mural cell pericytes, BBB damage can serve as an early biomarker for human cognitive decline that is independent of Aβ, and it has been supported by a study on regional BBB permeability using dynamic contrast-enhanced MRI (Montagne et al., 2015; van de Haar et al., 2016; Nation et al., 2019).

### Biomarker 3: Genetic Mutations

Genetics might play a crucial role in elucidating the cellular and molecular mechanisms of CSVD, and thus the pathophysiology of its hereditary forms. CADASIL, CARASIL, and several other forms of CSVD have been discussed with regard to the genetic factors and their pathways. Therefore, cellular, molecular, and biochemical changes underlying CSVD can easily be assessed using animal models of these rare single-gene disorders. Increasing the number and variety of transgenic, induced mutants and naturally occurring animal models of genetic disease are vital to identifying new genes that are the root cause of the disease; then, allow better understanding of the cellular and molecular mechanisms of genetic diseases and elucidating the genes involved in such diseases with complex inheritance patterns.

Besides that, CADASIL is a neurological syndrome characterized by CSVD, stroke, and vascular cognitive impairment and dementia caused by mutations in the *NOTCH3* gene (Joutel et al., 1996). A previous study showed that *NOTCH3* signaling is linked to vSMC coverage in retinal vessels and demonstrated that restoring *NOTCH3* signaling via genetic rescue and using a *NOTCH3* agonist antibody (A13) prevents the CSVD phenotype in both mouse models of CADASIL and *NOTCH3* knockout mice (Machuca-Parra et al., 2017). To date, four mutant mouse models express common CADASIL mutations: R90C, R169C, C428S, and R142C have been developed and studied in detail (Joutel et al., 1997). These models differ in their transgenic strategy and expression levels, endogenous *NOTCH3* expression, and the predicted effects of mutations on Notch function. Overall, from the mutant mouse models, data suggest that one or all these mechanisms may contribute to or modulate the phenotype, possibly explaining some of the clinical heterogeneity in CADASIL (Ayata, 2010).

### Biomarker 4: Systemic and Circulating Markers

#### Systemic Markers of Endothelial Dysfunction

The elevated level of endothelial dysfunction biomarkers in the blood of patients with CSVD had provided evidence of the involvement of EC failure in the pathomechanism of CSVD. Endothelial dysfunction may have multiple pathological pathways; however, most research focuses only on one pathway to study the circulating biomarker; therefore, it is important to consider multiple biomarkers of different pathways related to endothelial dysfunction so as to provide a greater opportunity to understand the disease mechanism and to eventually develop prevention and therapeutic approaches.

The first and major systemic biomarkers for endothelial dysfunction are inflammation markers. Inflammatory biomarkers of endothelial dysfunction, such as C-reactive protein (CRP), interleukin-6 (IL-6), intracellular adhesion molecule-1 (ICAM1), and *E*-selectin, have been widely studied and associated with CSVD in human and animal models (Cuadrado-Godia et al., 2018; Gu et al., 2019). The progression of WMHs in CSVD has been associated with higher expressions of ICAM1, CRP, and MMP9, thus supporting the role of endothelial dysfunction in CSVD (Markus et al., 2005; Satizabal et al., 2012; Kim et al., 2014; Gu et al., 2019). In addition, studies have also found associations between IL-6, E-selectin, and vascular cell adhesion molecule levels and the presence of microbleeds, lacunar infarcts, and WMHs (Rouhl et al., 2012; Gu et al., 2019). Therefore, enough evidence has shown that increased levels of different circulating inflammatory biomarkers were associated with the presence of different forms of CSVD, and levels of these biomarkers can be measured routinely in clinical and laboratory settings using plasma serum.

The second type are serum neurofilament (NfL) markers. Measuring the NfL level has been reported to be a direct approach of measuring the extent of neuronal damage (Uiterwijk et al., 2016). Since NfL is a crucial scaffolding protein in the neuronal cytoskeleton, the NfL released upon neuronal damage into the ECM, CSF, and blood can be a suggestive measure of endothelial dysfunction (Cuadrado-Godia et al., 2018); a previous study had reported that a high level of NfL is associated with the presence of a recent small subcortical infarct (Gattringer et al., 2017).

The next type of biomarkers are serum albumin (SA) and albuminuria markers. The leakage of albumin during BBB dysfunction is associated with increased BBB permeability, especially in the aging brain. Previous studies found that increased SA levels and the CSF/SA ratio are associated with the presence of WMHs in CSVD and vascular dementia, which serve as surrogate markers for BBB breakdown (Skoog et al., 1998; Simpson et al., 2007; Cuadrado-Godia et al., 2018). On the other hand, albuminuria has also been suggested as a marker for endothelial dysfunction (Stehouwer and Smulders, 2006), whereby multiple studies had supported that peripheral systemic microvascular disease marker, that is, albuminuria, is beneficial for the assessment of cerebral microvascular lesions and it had been associated with neuroimaging marker in CSVD (Cuadrado-Godia et al., 2018; Georgakis et al., 2018).

Another type of widely studied biomarkers of endothelial dysfunction are coagulation and hyperhomocysteinemia markers. One of the most crucial coagulation factors in circulation system is fibrinogen, which has been widely associated with endothelial dysfunction, followed by BBB damage, and is a beneficial marker for the disease (Bridges et al., 2014). Fibrinogen is a large plasma glycoprotein and its breakdown products are removed from cerebral tissue by the local plasminogen system or plasminogen activator. However, an increased plasma level of the tissue factor pathway inhibitor was associated with the presence of lacunar infarcts (Knottnerus et al., 2012). On the other hand, several studies had shown that higher levels of homocysteine in plasma serum were also associated with the presence of WMHs and SBIs (Vermeer et al., 2002; Sachdev et al., 2004; Kloppenborg et al., 2011).

#### Circulating Markers: Microparticles (MPs)

MPs are non-nucleated, small, and membrane-enclosed extracellular microvesicles (Dignat-George and Boulanger, 2011; Bebawy et al., 2013; Berezin, 2015). Their size ranges from 0.1 to 1 μm in diameter; they are particularly formed from membrane phospholipid exocytic blebs that are released from the cell surface by the proteolytic breakdown of the cytoskeleton due to cellular activation, injury, or apoptosis (Owens and Mackman, 2011; Nomura and Shimizu, 2015; Chiva-Blanch et al., 2016). The compositions of MPs are heterogeneous; they can be produced by many different cell types and characterized into subpopulations by the presence of cytoplasmic components and various surface antigens, which are characteristic of the state of the cell from which they originate and of the type of stimulus (Hussein et al., 2003; Lacroix et al., 2010; Burger and Touyz, 2012). Based on their cluster of differentiation, the MP subpopulation includes endothelial cell–derived MPs (EMPs): CD144, CD62E, or CD3; platelet-derived MPs (PDMPs): CD41a, CD42b, CD62P; red blood cell–derived MPs (RMPs): CD235a; and leukocyte-derived MPs (LMPs): CD45, CD4, CD8, and CD14 (Martinez et al., 2011; Andriantsitohaina et al., 2012).

MPs can serve as a procoagulant because they bear functionally bioactive phospholipids and cyto-adhesion molecules, such as phosphatidylserine and procoagulant protein tissue factor, that play major roles as cellular activators of the clotting cascade (Hussein et al., 2003; Owens and Mackman, 2011). Moreover, the formation of MPs might contribute to the disorganization of the proper function of endothelium layers. For example, Martinez et al. (2011) have shown that endothelial dysfunction caused by MPs lowered the production of NO and thus induced vascular inflammation that potentially contributed to the prothrombotic state within the arterial wall and propagated atherosclerosis, a hallmark of endothelial dysfunction. Besides, this dysfunction is also demonstrated by the shedding of EMPs that express platelet EC adhesion molecule-1 (i.e., CD31) that has been implicated to feature in ischemic stroke subtypes (Grammas et al., 2011).

Apart from endothelial dysfunction, Schreiber et al. (2013) argued about another common pathomechanism of CSVD that is related to the disorganization of arterial segmental walls and luminal narrowing. These arose due to accumulations of MPs alongside cholesterol crystals that caused arteriolosclerosis, which may result in hypoperfusion that accompanied infarcts and WMHs (Ogata et al., 2011; Schreiber et al., 2013). To date, limited studies are available to implicate the role of MPs in thrombosis (Owens and Mackman, 2011) and their relationships with CSVD. However, there is evidence that MP levels are increased in patients with cardiovascular diseases and risk factors, including acute coronary syndromes, diabetes, hypertension, hypertriglyceridemia, and the spectrum of CSVD (Puddu et al., 2010; Cheng and Dong, 2012; Kanhai et al., 2014; Vilar-Bergua et al., 2016).

Finally, a major problem relates to the fact that CSVD has multiple features and measuring the MPs of blood samples does not necessarily correspond to what happens to all CSVD features; different MP subpopulations may have different microthrombogenic effects on the progression of CSVD, but it is clearly stated that the MP level increased gradually prior to CSVD. In conclusion, considerable evidence suggests that MPs may play an important role in CSVD, although many molecular details still need to be clarified. The use of comprehensive panels of circulating MP biomarkers exploring the functioning of the different biological pathways may be useful to study CSVD.

### Role of Experimental Animal Models to Validate Biomarkers for CSVD

In animal models, plasma and cerebrospinal fluid biomarkers can assist in the development and implementation of similar approaches in clinical populations. These biomarkers may also be helpful in decisions for an advance treatment to human testing. Longitudinal studies in animal models can determine the initial presentation and progression of biomarkers that will be used to assess the disease-modifying efficacy of drugs. The refinement of biomarker approaches in preclinical systems will not only aid in drug development but may also facilitate diagnosis and disease monitoring (Sabbagh et al., 2013).

Furthermore, relationships in animal models can be investigated between peripheral biomarkers and readily available neuropathology; these can be translated into human studies where biomarkers are accessible, but neuropathology is often not. MicroRNAs (miRNAs) have also been implicated in disease pathogenesis and recommended as a putative biomarker (i.e., in patients with AD) (Wang et al., 2008, 2012). In non-transgenic mice fed a high-fat diet, reduced expression of multiple miRNAs was observed in the serum (Meissner et al., 2017). Substantial translational work is required before miRNAs can be used in the clinic; however, the approach is advancing rapidly.

In addition, since hypertension is one of the main risk factors for the development of CSVD, is a major contributor to stroke, and the most common cause of vascular dementia, chronic hypertensive rat models have been shown to bear similarities to most key features of CSVD. According to findings by a previous study, the mouse model of angiotensin II (AngII)-induced hypertension was an appropriate animal model for early onset CSVD and therefore, vascular cognitive impairment, pathologies commonly preceding vascular dementia (Meissner et al., 2017).

## Therapeutic Approaches in Csvd

### The Challenges

To date, a unifying pathomechanism of CSVD remains elusive, and hence contributes to the current lack of effective therapeutic strategies in preventing and treating CSVD (De Silva and Miller, 2016). Indeed, it is rather difficult to find clinical trials that focus specifically on different subtypes of CSVD as most available trials involve a mixture of small and large vessel diseases. Clinical trials testing for the efficacy of treatments are further challenged with the inability to directly visualize small vessels of interest using the standard neuroimaging techniques in the current routine clinical practice. Consequently, a trial might be mistakenly regarded as failed as the neuroimaging features of CSVD reflect irreversible pathological consequences of small vessel disease on the brain parenchyma, rather than the vascular changes itself (Zwanenburg and van Osch, 2017). Besides, the lack of animal models that replicate all aspects of clinical CSVD in humans add barriers in the pursuit of elucidating effective treatment and preventive interventions (Hainsworth and Markus, 2008).

### The Current Perspectives

Thus far, targeting the risk factors of diseases such as hypertension and hypercholesterolemia remains the focus of therapeutic approaches in CSVD despite its controversial long-term outcomes (Shi and Wardlaw, 2016). Here, we outline several therapeutic approaches commonly used in CSVD: the anti-hypertensive, anti-hyperlipidemic, and anti-platelet agents.

#### Anti-hypertensive Agents

In line with human studies, SHRSP rats demonstrate similar histopathological changes such as arteriosclerosis and lipohyalinosis of small vessels (Lammie, 2000; Grinberg and Thal, 2010; Schreiber et al., 2013; Ogata et al., 2014). Therefore, it is plausible that anti-hypertensive treatment might be able to protect the brain from further damage by attenuating the vicious cycle of increasing blood pressure and progressive arterial damage. Evidently, anti-hypertensive treatment in the SHRSP rat model has been found to halt the development of fibroid necrosis in cerebral arterioles (Richer et al., 1997) and preserve endothelial function (Krenek et al., 2001), in addition to its blood pressure–lowering effects (Richer et al., 1997; Krenek et al., 2001).

In clinical studies, available data suggest conflicting results in terms of the efficacy of anti-hypertensive treatment in reducing the rate of WMH progression in patients with stroke – ranging from a significant reduction to no effects (Dufouil et al., 2005; Weber et al., 2012; Bath and Wardlaw, 2015; De Silva and Miller, 2016). Nevertheless, a recent meta-analysis had concluded that anti-hypertensive treatments delay the progression of WMHs in CSVD, while no effects were found on brain atrophy (van Middelaar et al., 2018). Besides, anti-hypertensive treatment had been shown to significantly decrease the risk of lacunar stroke in elderly patients with isolated systolic hypertension (Perry et al., 2000). However, trials investigating the role of anti-hypertensive treatment as a secondary prevention of stroke, which included patients with lacunar stroke, yielded contradictory findings (White et al., 2015). Data pertaining to the optimal timing of anti-hypertensive treatment in CSVD and its effects on other neuroimaging markers of CSVD, such as microbleeds, lacunes, acute small subcortical infarcts, and enlarged perivascular space, are scarce and should be sought in further research (van Middelaar et al., 2018).

#### Anti-hyperlipidemic Agents

Statin, an HMG-CoA reductase inhibitor, is prescribed principally as an anti-hyperlipidemic agent in CSVD. Research evidence suggests that statin treatment might be beneficial in terms of preventing stroke recurrence in patients with small vessel stroke (Amarenco et al., 2009). Linear to this finding, pleiotropic effects of statins have been observed in several experimental animal models of CSVD. A marked reduction of brain expression of inflammatory markers and increased endothelial NO-synthase expression in carotid arteries were noted upon treatment with rosuvastatin in SHRSP rats (Gelosa et al., 2010).

In another study involving a similar stroke-prone model, rosuvastatin treatment was found to attenuate renal inflammatory processes and to delay the onset of brain damage (Sironi et al., 2005). In hyperhomocysteinemic rats, simvastatin treatment was found to inhibit homocysteine-induced CRP generation in vSMCs, hence contributing to reduced vascular inflammatory responses (Pang et al., 2016). Recent findings have also revealed that simvastatin treatment produced more pronounce effects than atorvastatin treatment in ameliorating oxidative stress in hyperhomocysteinemic rats (Nikolic et al., 2017).

Clinical evidence suggested that pre-stroke statin administration reduces WMH progression, improves executive function (Xiong et al., 2014), and promotes better functional outcomes upon discharge in patients with ischemic stroke (Martínez-Sánchez et al., 2009). Consistent with these findings, a recent study reported that a low-dose rosuvastatin treatment delayed WMH progression in elderly patients with hypertension (Ji et al., 2018). Nonetheless, despite the numerous beneficial effects exhibited by statin, aggressive anti-hyperlipidemic treatment in patients with stroke requires specific consideration in view of the increased risk of hemorrhagic stroke (Goldstein et al., 2008).

#### Anti-platelet Agents

The use of anti-platelet agents remains one of the strategies in secondary stroke prevention after lacunar stroke (Nakajima et al., 2014). In general, an anti-platelet agent maintains the patency of blood vessels by inhibiting platelet aggregation and thrombus formation. In SHRSP rats, both clopidogrel and cilostazol had shown superior effects as compared with aspirin in terms of spontaneous infarct volume reduction (Omote et al., 2012). However, only the cilostazol-treated group had improved cognitive and motor functions. Concurrently, cilostazol increased both insulin-like growth factor type 1 receptor (IGF-1R) positive ratio and IGF-1Rβ expression in the hippocampus (Omote et al., 2012). Importantly, IGF-1 had been previously shown to increase neurogenesis in the same brain region (Åberg et al., 2000). In a similar rodent model, both terutroban and aspirin were found to attenuate the expression of neuroinflammatory markers and preserve vascular reactivity in the carotid arteries. The endothelial protective effects were more pronounced in the group treated with terutroban (Gelosa et al., 2010). In the CCH rat model, dipyridamole exerted neuroprotective effects by reversing or slowing the progression of several pathophysiological changes found commonly in the rat model (Lana et al., 2014, 2017). Mirroring these effects, dipyridamole had been found to restore spatial working memory in the same rat model (Melani et al., 2010).

A pooled analysis of randomized trials had supported the use of single anti-platelet agents as a secondary prevention modality following lacunar stroke (Kwok et al., 2015). Nonetheless, the prolonged use of dual anti-platelet agents incites a therapeutic dilemma in view of the increased risk of cerebral hemorrhage. Specifically, a randomized controlled trial, the Secondary Prevention of Small Subcortical stroke Study (SPS3) had found that dual anti-platelet treatment with aspirin and clopidogrel in patients with lacunar stroke significantly increased the risk of major hemorrhage and mortality, while no beneficial effect was found in terms of reducing the risk of recurrent stroke (The SPS3 Investigators, 2012). Similarly, in the Management of Atherothrombosis with Clopidogrel in High risk patients (MATCH) trial, which involved patients with recent ischemic stroke or transient ischemic attack, the aspirin and clopidogrel combination had been shown to increase the risk of major hemorrhage as compared to clopidogrel alone (Diener et al., 2004).

### Future Direction of Therapeutic Approaches in CSVD

There has been a recent proposition to view CSVD as a dynamic whole-brain disease to guide on new direction of therapeutic approaches in CSVD (Shi and Wardlaw, 2016). Nevertheless, any new therapeutic strategy must address its potential in treating the root cause of the disease, reversing both clinical and pathological signs of the disease, and halting disease progression. Perhaps a better picture of the pathomechanism and therapeutic approaches in CSVD could be sought in animal models by interpreting the neuroimaging findings together with neurobehavioral manifestations and physiological changes noted at the molecular level. In addition, the administration of a combination of drugs or drugs that have multiple modes of action (Bath and Wardlaw, 2015) together with a non-pharmacological approach might be necessary to modulate different pathways involved in the disease process. Besides, the financial impact, route of administration of drugs, and adverse reactions following long-term treatment should be critically appraised in future studies in view of the chronic nature of CSVD (Bath and Wardlaw, 2015). Here, we highlight several lines of plausible emerging strategies worth considering for CSVD therapeutic approaches.

#### Combating Oxidative Stress

Recently, compelling evidence from experimental models suggests that oxidative stress might be one of the key players involved in the development of arteriopathy in CSVD (De Silva and Miller, 2016) and cerebrovascular changes observed in neurodegenerative diseases (Carvalho and Moreira, 2018). Coincidentally, several drugs that were conventionally used as a therapeutic approach in CSVD exhibit antioxidant properties (Nikolic et al., 2017). A growing body of literature has investigated the role of antioxidants as a therapeutic modality in the experimental animal model of CSVD (Chen et al., 2001; Bagi et al., 2003; Sasaki et al., 2011; Ueno et al., 2015; Guan et al., 2018).

Several authors had found that natural antioxidants, such as vitamin E, ascorbic acid, L-carnitine, astaxanthin, and nigella sativa supplementation, reduce oxidative stress in SHRSP and CCH rat models, as depicted by increased activities of superoxide dismutase, catalase, and glutathione in the brain (Guan et al., 2018), plasma total antioxidant status (Chen et al., 2001), and vascular superoxide dismutase (Chen et al., 2001). The amelioration of oxidative stress was also evidenced by reduced vascular nicotinamide adenine dinucleotide phosphate (NADPH) oxidase (Chen et al., 2001), serum 8-isoprostane (Bagi et al., 2003), urinary 8-hydroxy-2’-deoxyguanosine (Sasaki et al., 2011), lipid peroxidation of oligodendrocytes (Ueno et al., 2015), plasma malondialdehyde level (Murad et al., 2014; Guan et al., 2018), and malondialdehyde level in the brain (Guan et al., 2018).

Together with the oxidative stress–lowering effect, vitamin E or its compounds as supplements have been found to prevent neuronal death (Tagami et al., 1999; Annaházi et al., 2007; Yamagata et al., 2010), preserve the structure of the hippocampus (Murad et al., 2014), decrease cerebral thrombotic tendency (Noguchi et al., 2001), improve vascular function (Chen et al., 2001), prevent the progression of hypertension (Chen et al., 2001; Noguchi et al., 2001), and improve cognition (Murad et al., 2014) in SHRSP and CCH rat models.

On the other hand, a combination of α-tocopherol and lovastatin treatment in SHRSP had led to similar findings in terms of reduced oxidative stress, preservation of hippocampal structure, and improved cognition (Guimarães et al., 2015). It is therefore possible that the observed beneficial effects were partly contributed by the capability of vitamin E or its compounds in reducing free radicals and ROS that were generated in these rat models. Recently, a randomized double-blind placebo-controlled trial demonstrated that 2 years of mixed tocotrienol supplementation successfully halted the progression of white matter lesions in the subjects who presented with cardiovascular risk factors (Gopalan et al., 2014). In view of its tolerability and availability as a natural antioxidant, it was proposed that it could be used as a long-term supplement in individuals with ischemic white matter damage (Gopalan et al., 2014). Notably, research evidence had shown that a very high dose of α-tocopherol supplementation increased blood pressure and adversely altered the hippocampal structure in SHRSP rats (Miyamoto et al., 2009). Therefore, further temptations to use high doses of vitamin E or its compounds in the clinical setting should be resisted until further clarification.

Concurrent with the attenuated oxidative stress level, vitamin C, astaxanthin, and thymoquinone supplements retard the progression of hypertension in SHRSP and hyperhomocysteinemic rat models (Chen et al., 2001; Bagi et al., 2003; Sasaki et al., 2011; Guan et al., 2018). Extra beneficial effects in terms of the inhibition of cerebral vascular thrombosis were noted in the astaxanthin-supplemented SHRSP group (Sasaki et al., 2011). It was postulated that both effects observed in the astaxanthin-supplemented group were related to the increased bio-availability of NO secondary to the reduction of the ROS inhibitory action on the NO (Sasaki et al., 2011). Meanwhile, L-carnitine supplements in CCH rats significantly reduced the pathological hallmarks of ischemic white matter disease by enhancing the myelin sheath thickness and oligodendrocyte marker expression (Ueno et al., 2015). Thymoquinone supplements had been shown to reduce neuroinflammation in the SHRSP model, as evidenced by the decreased miRNA expression of IL-1β, IL-6, monocyte chemoattractant protein-1, and cyclooxygenase-2 (COX-2) in the brain (Guan et al., 2018).

A few studies had demonstrated that superoxide dismutase mimetic (e.g., tempol), an antioxidant enzyme, alleviates oxidative stress in the SHRSP, as measured by the increased plasma total antioxidant status and reduced vascular superoxide anions (Park et al., 2002). These changes were accompanied by the attenuation of vascular remodeling (Park et al., 2002). Importantly, a recent study in advanced-stage SHRSP had discovered that tempol failed to reduce blood pressure effectively and, in fact, aggravated the preexisting renal injury (Sugama et al., 2014). Notably, the destructive effects observed in the kidney were not seen in hydralazine-treated group, which had demonstrated a significantly lower blood pressure than the untreated group (Sugama et al., 2014).

Considering the role of oxidative stress in the pathomechanism of CSVD, combating oxidative stress might be a promising therapeutic approach. Lessons from vascular disease and/or stroke trials indicated that antioxidant treatment might fail to show benefits in these settings in view of the limitation of the study design and failure of the relevant antioxidant to adequately reverse oxidative stress, which might be particularly true in advanced stages of the disease (Pong, 2003; Drummond et al., 2011). In future studies, adverse outcomes following long-term treatment should be monitored closely since ROS plays an important physiological role in maintaining cerebral vascular function (De Silva and Miller, 2016).

#### Modulation of Cyclic Adenosine Monophosphate (CAMP) System

Cilostazol is a specific phosphodiesterase III inhibitor that has been shown to prevent platelet aggregation, reduce the oxidative stress level, and exert vasodilatory effects by increasing the intracellular level of CAMP and endothelial NO-synthase activity (Omote et al., 2012). Cilostazol treatment in the SHRSP and CCH rat models have been found to reduce oligodendrocyte cell death (Lee et al., 2006, 2007; Watanabe et al., 2006; Miyamoto et al., 2010), spontaneous infarct volume (Omote et al., 2012), BBB permeability (Edrissi et al., 2016), and microglial activation, which is a major source of inflammatory cytokines (Watanabe et al., 2006; Edrissi et al., 2016). In line with these findings, several studies noted that cilostazol treatment lowered the production of tumor necrosis factor-α production, a marker of the proapoptotic protein, and suppressed the accumulation of 4-hydroxy-2-nonenal-modified protein, which is a marker of oxidative neuronal damage that appeared post ischemic/reperfusion injury (Watanabe et al., 2006). Meanwhile, prolonged cilostazol treatment in SHRSP rats resulted in significantly attenuated vascular wall thickening, perivascular fibrosis, microglial activation, and the degree of white matter lesions (Fujita et al., 2008).

The reduction of oligodendrocyte death and regeneration of white matter that were observed in the cilostazol-treated group diminished upon the inhibition of protein A/K, which modulates the phosphodiesterase inhibition (Miyamoto et al., 2010). This suggests that the inhibition of phosphodiesterase III has a potential benefit in terms of increasing oligodendrogenesis and remyelination of the damaged white matter area (Miyamoto et al., 2010). Several observed beneficial effects were associated with improved spatial learning memory (Miyamoto et al., 2010) and motor function (Edrissi et al., 2016) in the cilostazol-treated group. A few studies proposed that the neuroprotective effects of these drugs were attributed to the enhanced cAMP-responsive element-binding protein phosphorylation signaling pathway (Watanabe et al., 2006; Lee et al., 2007) and subsequent activation of Bcl-2 and COX-2 (Watanabe et al., 2006) in the cilostazol-treated group. Several authors suggested that the observed improvements in terms of the cognitive function might be partly accounted for by the increased vascular endothelial growth factor receptor 2 expression in the peri-infarct area (Omote et al., 2014) and increased IGF-1Rβ-positive cells in the hippocampus, which might be beneficial in terms of enhancing neuroplasticity, neurotransmission, and neurogenesis (Omote et al., 2012).

Indeed, the pleiotropic effects of cilostazol had attracted several researchers to investigate its practicality in the clinical setting of CSVD. In a multicenter, randomized, double-blind, placebo-controlled trial, cilostazol treatment in patients with acute lacunar infarction had resulted in a favorable decrease in the pulsatility index in comparison to the placebo-treated group (Han et al., 2013). Subgroup analysis from the same study revealed that cilostazol decreased cerebral arterial pulsatility in patients with mild WMH changes (Han et al., 2014). Meanwhile, in a pilot study involving patients with recent small subcortical infarcts, it was suggested that early treatment with cilostazol might reduce the plasma inflammatory biomarkers, which is probably associated with poor neurological outcomes (Saji et al., 2018). In line with these findings, cilostazol treatment of patients with acute stroke with small vessel occlusion had resulted in a shortened length of hospital stay and better neurological outcomes (Nakase et al., 2013).

#### Neurotrophins

The role of cerebrolysin as a neuroprotective and neurotrophic compound has been investigated in various experimental animal models and clinical studies of stroke and neurodegenerative disease (Rockenstein et al., 2003; Gauthier et al., 2015; Liu et al., 2017; Zhang et al., 2017). However, the value of cerebrolysin as a candidate for the treatment of CSVD remains inadequately explored. In a recent development, cerebrolysin treatment in a CCH rat model resulted in the increased expression of plasticity-related synaptic proteins concurrent with significant improvements in terms of the cognitive function (Liu et al., 2017). Notably, a review of randomized controlled trials had concluded that cerebrolysin might exert a positive effect on the cognitive function of patients with mild to moderate vascular dementia (Chen et al., 2013). This implicates that cerebrolysin might be a worthy candidate for further research in CSVD.

### Other Potential Non-pharmacological Therapeutic Approaches

Other potential therapeutic approaches, such as smoking cessation, reduced salt intake, fasting, and increased physical activity, might be of benefit in the modification of CSVD risk factors, which are common to most non-communicable cardio-cerebrovascular diseases. In the CCH rat model, physical exercise had been shown to confer protection against BBB impairment (Lee et al., 2017), reduce the oxidative stress level (Cechetti et al., 2012), promote neurogenesis, and increase the hippocampal mature brain-derived neurotrophic factor level (Choi et al., 2016). Concurrent cognitive function improvement was noted in these studies (Cechetti et al., 2012; Choi et al., 2016; Lee et al., 2017). Early intervention with physical exercise after the BCCAO procedure in the CCH rat model significantly reduced cerebral microvascular inflammation, as evidenced by the decreased cerebral NADPH oxidase gene expression and endothelial–leukocyte interactions (Leardini-tristão et al., 2016). In support of these findings, a recent review had concluded that exercise increases the bioavailability of neurotrophins, protects blood vessels from damage, and reduces vascular disease risk factors in patients with subcortical ischemia. Apart from these, it was noted that exercise improves the cognitive function in this subset of patients (Dao et al., 2018).

Besides, intermittent fasting pretreatment prior to BCCAO in the CCH rat model has been shown to alleviate oxidative stress in the hippocampus, as evidenced by decreased malondialdehyde concentrations, increased glutathione concentrations, higher gene expression of antioxidative enzymes, and enhanced superoxide dismutase activity as compared to the non-fasting group (Hu et al., 2017). In fact, it has also been found to mitigate neuroinflammation in the hippocampus, which was demonstrated by the presence of lower microglia density and the expression of inflammatory proteins. These changes were accompanied by protective effects on the cognitive function (Hu et al., 2017).

In addition, the role of dietary salt restriction in CSVD should be further investigated in view of the evidence that high dietary salt intake in SHRSP rats exacerbated hypertension and increased the mortality rate (Chen et al., 1997). The need for further research in this field is supported by a recent clinical finding that had shown that patients with stroke with long-term higher dietary salt intake have a higher possibility of developing CVSD (Makin et al., 2017).

All in all, it is critical to carefully evaluate the risk and benefits of each treatment based on each patient. The apparent disparity in terms of the findings might be a reflection of the dynamicity and complexity of the disease in which different pathomechanisms are involved in different disease subtypes. Indeed, more studies are required to find a novel approach in CSVD treatment as a step toward establishing clinical practice guidelines of CSVD management. Therefore, several experimental animal models of CSVD should be used to discern the treatment effects on different pathological features of the disease. Large-scale randomized control trials that accurately identify the subtypes and stages of CSVD are required to assess the effectiveness of these treatments in preventing CSVD development in high-risk subject as well as in halting disease progression. Further studies should also be conducted to elucidate the impact of systematically incorporating non-pharmacological therapeutic strategies in the current management of CSVD. Nonetheless, it should be kept in mind that a safe, tolerable, and non-invasive treatment would be critical to increase the compliance rate in patients, which is one of the keys to successful treatment.

## Conclusion

Cerebral small vessel disease is a relatively heterogenous disease process and an important precursor to cognitive decline, stroke, and age-related functional decline. Despite the increasing number of research studies on CSVD, the pathomechanism in terms of its vascular pathology and brain injury remain elusive with various contentions on management and prevention. However, current technological advances in elucidating the disease pathomechanism may increase our understanding of the natural history of CSVD up to the molecular level. The main hurdle in exploring the natural history of CSVD is the fact that there is a coexistence of multiple forms, such as WMHs, lacunar infarcts, and microbleeds. Therefore, more attention and targeted efforts are needed to better disentangle the clinical consequences of CSVD. Meanwhile, advanced experimental and clinical trials are crucial to better elucidate the diagnostic criteria of CSVD and provide better therapeutic and preventive measures to halt and reduce the burden of disability, AD, or dementia caused by CSVD; the establishment of correct animal models to study the specific pathogenesis and mechanism of multiple forms of CSVD is highly beneficial.

## Author Contributions

MM conceived, reviewed, and revised the final content of the manuscript. CN drafted the initial and original content of the manuscript and revised it. NA, AS, and MG contributed equally to drafting the initial and original content of the manuscript. All authors have jointly agreed on the final draft for submission.

## Conflict of Interest

The authors declare that the research was conducted in the absence of any commercial or financial relationships that could be construed as a potential conflict of interest.
